# Evaluation of Retinal Vessel Morphology in Patients with Parkinson's Disease Using Optical Coherence Tomography

**DOI:** 10.1371/journal.pone.0161136

**Published:** 2016-08-15

**Authors:** Robert Kromer, Carsten Buhmann, Ute Hidding, Matthias Keserü, Diana Keserü, Andrea Hassenstein, Birthe Stemplewitz

**Affiliations:** 1 Department of Ophthalmology, Hamburg Medical Center Hamburg-Eppendorf, Hamburg, Germany; 2 Department of Neurology, Hamburg Medical Center Hamburg-Eppendorf, Hamburg, Germany; Heinrich-Heine-Universitat Dusseldorf, GERMANY

## Abstract

**Purpose:**

The retina has been found affected in Parkinson’s disease (PD). It is unclear if this is due to neurodegeneration of local dopamine-dependent retinal cells, a result of central nervous degeneration including the optic nerve or retinal small vessel disease. This study aimed to detect changes of the retinal vasculature in PD patients compared to controls.

**Methods:**

We examined 49 PD patients and 49 age- and sex-matched healthy controls by spectral domain optical coherence tomography (SD-OCT) with a circular scan centred at the optic disc. Vessels within the retinal nerve fibre layer were identified by an automated algorithm and thereafter manually labelled as artery or vein. Layer segmentation, vessel lumen and direct surrounding tissue were marked automatically with a grey value and the contrast between both values in relation to the surrounding tissue was calculated. The differences in these grey value ratios among subjects were determined and used as an indicator for differences in vessel morphology. Furthermore, the diameters of the veins and arteries were measured and then compared between the groups.

**Results:**

The contrast of retinal veins was significantly lower in PD patients compared to controls, which indicates changes in vessel morphology in PD. The contrast of arteries was not significantly different. Disease duration, disease stage according to Hoehn and Yahr or age did not influence the grey value ratios in PD patients. Vessel diameter in either veins or arteries did not differ between subject groups. The contrast of retinal veins contralateral to the clinically predominant and first affected side was significantly lower compared to the ipsilateral side.

**Conclusion:**

Our data show a potential difference of the retinal vasculature in PD patients compared to controls. Vascular changes in the retina of PD patients might contribute to vision-related complaints in PD.

## Introduction

Parkinson’s disease (PD) is the second common central neurodegenerative disorder and characterised by the loss of dopaminergic neurones in the central nervous system [1]. Neurodegeneration and accumulation of proteinaceous cytoplasmic inclusions, so-called Lewy bodies, are the main underlying histopathologies. Cell degeneration and dopamine depletion in PD cause motor and non-motor symptoms, including impairment of the autonomic nervous system, the olfactory system and the visual system [2,3]. The underlying aetiology of visual symptoms such as reading difficulties, diplopia or changes in contrast vision, which are frequently reported by PD patients [4], is still not well understood.

The retina possesses the unique advantage that vessels are directly visible and microcirculation can be imaged *in vivo*. This provides the opportunity to detect and monitor ocular and systemic diseases. An analysis of the retinal microvasculature provides information about both the structure and the function of the vessels [5]. Several groups, including our own, have used optical coherence tomography (OCT) [6–12] and scanning laser polarimetry (SLP) [12] to investigate degenerative changes of the retina in PD patients. While some of these studies found reductions in the retinal nerve fibre layer (RNFL) and the macular volume, as well as thinning of several retinal layers and thickening of others, other groups have described unchanged findings in PD patients compared to healthy controls [13,14]. Interestingly, as far as we know, retinal vasculature in PD has not yet been examined although small vessel pathology has been found to play a role in PD in the central [15–17] and peripheral nervous system [18–22]. Therefore, we investigated potential changes in retinal vessel morphology in PD patients compared to controls. We used spectral domain optical coherence tomography (SD-OCT), which is an established image-modality that yields non-invasive high-resolution cross-sectional images of biological tissue [23]. This technology is used in ophthalmology for diagnostic purposes as it allows the collection of *in vivo* information. Since this technique permits the display of tiny details of the retinal structure [24], it has become one of the most important imaging tools in diagnosing retinal diseases. The optical beam that is partly reflected by the sub-surface features of the tissue is collected. Nevertheless, most of the light is not reflected but scatters off the tissue at large angles. In SD-OCT the optical path lengths of received photons are recorded and allow rejection of those photons that scatter multiple times before detection. It is well known that tissue composition affects its light scattering properties [25].

**Fig 1:**
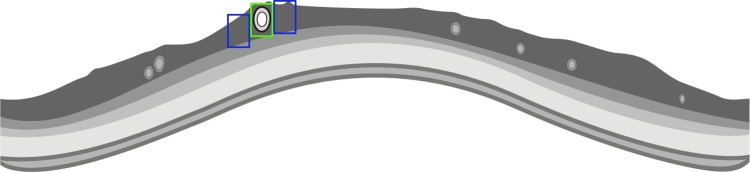
Schematic illustration of the SD-OCT-based analysis. The approaching light is reflected and scattered by the vessels. The hypothesis is that the characteristics of the change in contrast resemble the vessel morphology. In this image the simplified calculation of the change in grey value is achieved by comparing the mean grey value of the blue boxes with that of the green box.

We introduce a novel morphological method of using SD-OCT to analyse retinal vessel architecture. The grey value (signal strength) within the vessel lumen in the RNFL (where the vessel is located) is compared with its directly surrounding tissue in the same layer (see Fig 1). This is done in PD patients and healthy controls in search of an indicator for differences in vessel morphology separately for arteries and veins between the two groups.

## Subjects and Methods

### Subjects

We performed a prospective, cross-sectional study in 49 patients (mean age 64.1 years; age range 46–77 years, 29 males and 20 females) suffering from Parkinson´s disease (PD) according to UK Brain Bank criteria [26] and who had been diagnosed by a specialist in movement disorders (CB or UH) at the local neurological department. Disease severity was assessed according to Hoehn and Yahr [27], and the dominant side of disease symptoms was documented. The median disease duration was 8.1 years (range 3–25 years). Patients with atypical or other parkinsonian syndromes were excluded.

A total of 49 age- and sex-matched ophthalmologically and neurologically healthy subjects (mean age 63.9 years; age range 45–77 years, 29 males and 20 females) served as controls.

The 49 patients and 49 matched controls were selected from a collective of 108 PD patients and 165 healthy controls examined with spectral domain optical coherence tomography (SD-OCT). The selection was based firstly on sufficient OCT RNFL thickness data, which were available in 96 out of the 108 PD patients. Secondly, OCT scans had to fulfil consensus criteria for retinal OCT quality assessment (OSCAR-IB) to improve comparability and quality management of OCT-images [28]. Forty-nine out of the 96 patients (51%) met these criteria and were included in the study. As controls, 49 age- and sex-matched healthy subjects were chosen from a collective described in 2 previous studies concerning retinal and RNFL thickness [11,12]. The thickness of the RNFL around the optic nerve head was proven to be not different between included PD patients and controls [11,12] by application of two different OCT devices (Spectralis, Heidelberg Engineering and Cirrus, Zeiss).

Patients and controls were examined by an ophthalmic specialist (RK or BS) to exclude subjects with current or past ophthalmological diseases, such as glaucoma, age-related macular degeneration, diabetic or hypertensive retinopathy, ocular inflammation or patients who had retinal surgery. Furthermore, subjects with conditions that could potentially affect the RNFL or vessel morphology, such as normal tension glaucoma (NTG) or open-angle glaucoma, were excluded from the study.

Patients underwent a series of ophthalmic examinations including (i) slit lamp-assisted biomicroscopy of the anterior and posterior segment with non-dilated pupils to exclude ocular pathologies, (ii) non-contact tonometry (Nidek Tonometer NT-530) to exclude non-detected glaucoma, and (iii) SD-OCT image acquisition (SPECTRALIS; Heidelberg Engineering, 4^th^ Generation, Heidelberg, Germany, scan software version 1.7.0.0, analysis software version 5.4.6).

The research protocol followed the recommendations of the Declaration of Helsinki (Seventh revision, 64th Meeting, Fortaleza, Brazil), Good Clinical Practice and was approved by the ethics committee of the Hamburg Medical Council. Written informed consent was obtained from each patient and control subjects before they were entered into the study.

### Spectral Domain Optical Coherence Tomography (SD-OCT)

The RNFL scans around the optic nerve head were obtained applying non-contact frames in high resolution of the RNFL. The device is a combination of conventional OCT technology and cSLO. A superluminescent diode was used to emit a light beam with a wavelength of 870 nm. A reference image was taken with cSLO, linked and saved to the SD-OCT scan with an eye tracking system (TrueTrack^TM^, Heidelberg Engineering, Heidelberg, Germany). The automatic real-time averaging mode (ART) where multiple images of SD-OCT frames were averaged resulted in achieving even higher quality. In this study, only high-quality data with a total of at least 18 frames were used to provide images with low noise (high-resolution mode). Due to high-resolution scans, the individual layer of the RNFL was discriminable even in the absence of pupil dilatation. We first positioned the scan perfectly centred around the optic disc (3.4mm ring scan, RNFL-Nsite) and enabled the ART mode. To minimise variability, a single observer obtained three high-resolution scans for each patient. All images had to meet the following criteria of quality to be included in the study: (i) a clear fundus before and during image acquisition, (ii) absent scan and algorithm failures, (iii) consistent grey scale saturation of each RNFL with the retinal pigment epithelium showing maximal shading, and (iv) no discontinuity of the scanned layer. For vessel analysis, only peripapillary scans were used. This led to the standardised acquisition of as many vessels as possible in one scan and good comparability.

### Vessel Analysis

To investigate retinal vessels in PD patients and controls, we have introduced a new morphological method using SD-OCT for retinal vessel analysis. The position of vessels was detectable with high reliability in the SD-OCT scan. The grey value inside the vessel lumen (signal strength) was measured and compared to the grey of its direct surrounding tissue (compare Fig 1).

The SD-OCT-based vessel analysis was completed automatically to ensure reproducibility and facilitate independence from observer bias (except for vessel labelling). It was completed in several steps:

(1) **Detection of retinal vessels in the SD-OCT image.** The retinal blood vessels were detected based on the observation that the blood vessel sections were usually much brighter than the neighbouring non-vessel sections. The blood vessel sections within OCT images could thus be located through an iterative polynomial smoothing procedure proposed by Lu et al. [29]. Vessels diameters were recorded in pixels.
(2) **Manual labelling of retinal vessels in the cSLO.** A discrimination between arteries and veins increases the validity of detecting vascular differences between healthy subjects and patients. We (RK) labelled the automatically detected vessels from the SD-OCT scan using the accompanying cSLO image by applying the criteria proposed by Motte et al. [30]. All labelling was done using the implemented graphical interface.
(3) **Layer segmentation of the SD-OCT image.** We used the proposed consensus nomenclature of the International Nomenclature for Optical Coherence Tomography [IN_OCT] Panel [31] to classify the retinal layers and bands of a normal eye visible on SD-OCT images. Two boundaries were detected: the internal limiting membrane (ILM) and the boundary between the RNFL and ganglion cell layer (GCL). It was, therefore, possible to identify the innermost layer, the RNFL, as the vessels are located within this layer.

We chose a median filter for noise suppression—adopted from Herzog et al. [32]—due to its simplicity and its property of preserving the important macrostructure of the image. This suppressed most of the speckle and homogenised the retina and choroid by destroying the underlying microstructure. The pixel value of the resulting gradient image was not normalised. Therefore, all pixel values were linearly re-mapped to the range 0 to 1, leading to a shade-corrected image with reduced background intensity variations and enhanced contrast.

The ILM was detected when analysing each column of the image matrix, supposing that *r* is a vector containing the position of white pixels in ascending order. We applied the following difference operation on *r* to calculate *r′*, where *i* = 1,2,…,*length*(*r*) − 1.

$$r^{\prime }(i)=r(i+1)-r(i)$$

In this setting, *index i* was considered to be the position of the ILM layer boundary. The RNFL/GCL was detected the same way when searching for the next change in pixel value below the ILM. The described calculation was repeated for each column of the image matrix, resulting in a curve for the ILM.

The SD-OCT images contained a significant amount of noise; therefore, the curve contains many local minima and maxima. Curve fitting-based regularisation, modified from Yang et al. [33], was used to smooth the curves.

**Fig 2:**
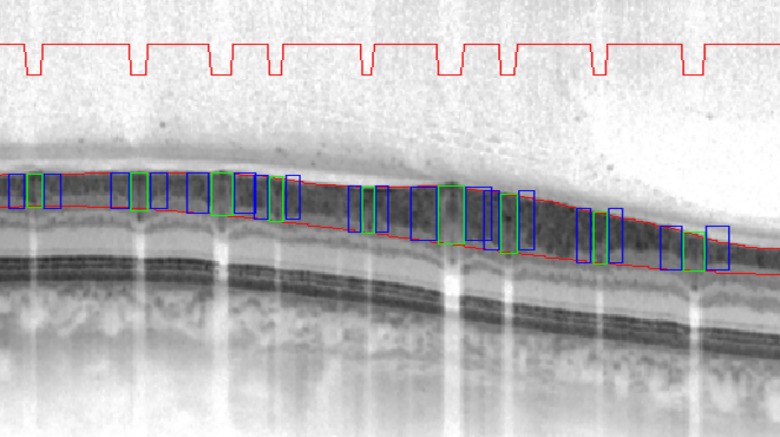
SD-OCT-based vessel analysis. The red line symbolises the detected vessels; the line drops when there is a vessel in the SD-OCT scan. The grey value of the vessel is measured, and depicted as the green box. This is compared to the grey value of the surrounding tissue, represented as blue boxes.

(4) **Analysis of vessel contrasts with their surrounding tissue.** The grey value vessel analysis was calculated as the quotient (ratio) of the difference of grey values of the lumen of the vessel and the surrounding tissue (contrast, depicted as green boxes in Fig 2) and the grey value of the surrounding tissue (represented as blue boxes in Fig 2).

$$vessel\;contrast\;analysis\;[\%]=\frac{grey\;value(vessel)-grey\;value(surrounding\;tissue)}{grey\;value(surrounding\;tissue)}*100$$

The resulting value is the change in contrast between the vessels and the surrounding tissue. It is necessary to build a ratio between contrast difference and surrounding grey vessel value since absolute values cannot be compared between patients owing to the varying contrast between differences (due to lens density, vitreous body density, eye axial length and internal scan processing). The intensity of a pixel is expressed within a given range between a minimum and a maximum, inclusive. This range is represented in an abstract way as a range from 0 (total absence, black) to 1 (total presence, white) with fractional values in between; higher values represent brighter pixels. Higher values in the analysis of the vessel contrast ratio, therefore, imply brighter vessels, a bigger difference between RNFL and vessels (RNFL is dark, compare Fig 2) and stronger scattering properties of the vessels.

Vessels were excluded where the mean grey value difference between left and right surrounding tissues was bigger than the change in brightness of the grey value of the vessel. By doing this, vessels that lie close to each other could be rejected to avoid their scattering properties interfering with each other. Further, manual correction was not performed. The mean vessel contrast for a vessel type (arteries or veins) was only calculated when at least two arteries or two veins were detected per scan.

All algorithms were implemented in MATLAB (version R2015b, The MathWorks Inc., Natick, Massachusetts, 2015) along with a graphical user interface.

### Statistical Analysis

Statistical analysis was performed using a commercially available software package (Prism 6 for Mac OSX; GraphPad Software, Version 6.0e). The means and standard deviations are presented. The *p*-values were corrected according to the Bonferroni method to correct for the performance of multiple statistical analyses. Paired parametric t-tests were used with two-tailed *p*-values. A *p*-value < 0.05 was considered to indicate statistical significance. The correlation was performed using Pearson correlation calculations, as the values sampled from the populations followed an approximate Gaussian distribution. The correlation coefficient is indicated by r. Presented box- and whiskers plots are mean values with 10–90% percentile lines. A single eye from each participant was randomly selected (using RAND function of Microsoft® Excel) and used for statistical analysis (phenotype). Patients and controls were compared in a matched pair analysis to control for possible influences of age and sex distribution. We also compared the inter-individual values of PD patients according to their dominant and non-dominant side of the disease. To look for possible correlations, the influence of age, disease duration and disease severity (assessed according to Hoehn and Yahr [27]) were also analysed.

## Results

**Fig 3:**
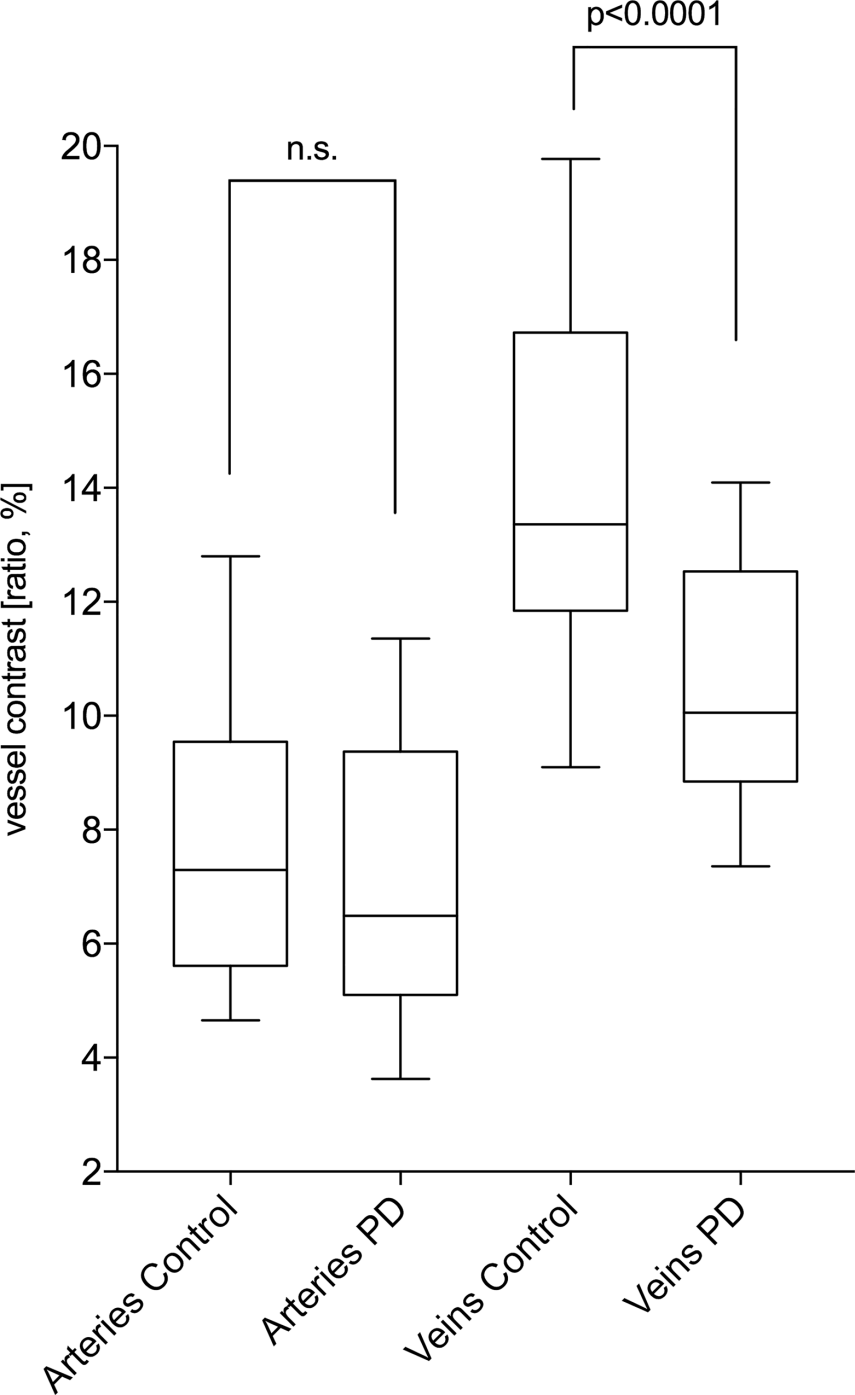
Comparison of the SD-OCT-based vessel analysis between patients with Parkinson’s disease (PD) and healthy control patients. Using a paired t-test, PD patients had a significantly lower grey value contrast for veins compared to controls (mean of differences = -3.877±4.464; *p*-value<0.0001). The grey value contrast for arteries was not different between the groups (*p*-value = 0.2926).

Our approach generated three averaged values for every vessel type: the grey value vessel ratio (grey contrast) as a parameter for vessel morphology—comparing the grey value within the lumen of the vessels to its surrounding tissue—, the mean vessel diameter and the number of detected vessels. Using a paired t-test, PD patients had a significantly lower grey value contrast for veins compared to controls (mean of differences = -3.877±4.464; *p*-value<0.0001; see Fig 3). The grey value contrast for arteries was not different between the groups (mean of differences = -0.7227±4.173; *p*-value = 0.2926; see Fig 3). The number of detected vessels (arteries or veins) was not different between the two groups as well (number of arteries: *p*-value = 0.0801; number of veins: *p*-value = 0.7072) which shows a comparable quality of the cSLO and OCT images in both groups, but we could detect slightly more veins than arteries, especially in the PD group (control patients: mean of differences = 0.4444±1.307; *p*-value = 0.0274; PD patients: mean of differences = 0.8936±1.165; *p*-value<0.0001).

The vessel width (in pixels) was significantly different between the arteries and veins (control patients: mean of differences = 2.276±1.632; *p*-value<0.0001; PD patients: mean of differences = 2.158±1.637; *p*-value<0.0001), however, it was not different between PD and control patients (arteries: mean of differences = -0.0679±1.649; *p*-value = 0.7766; veins: mean of differences = -0.2047±2.073; *p*-value = 0.5111).

**Fig 4:**
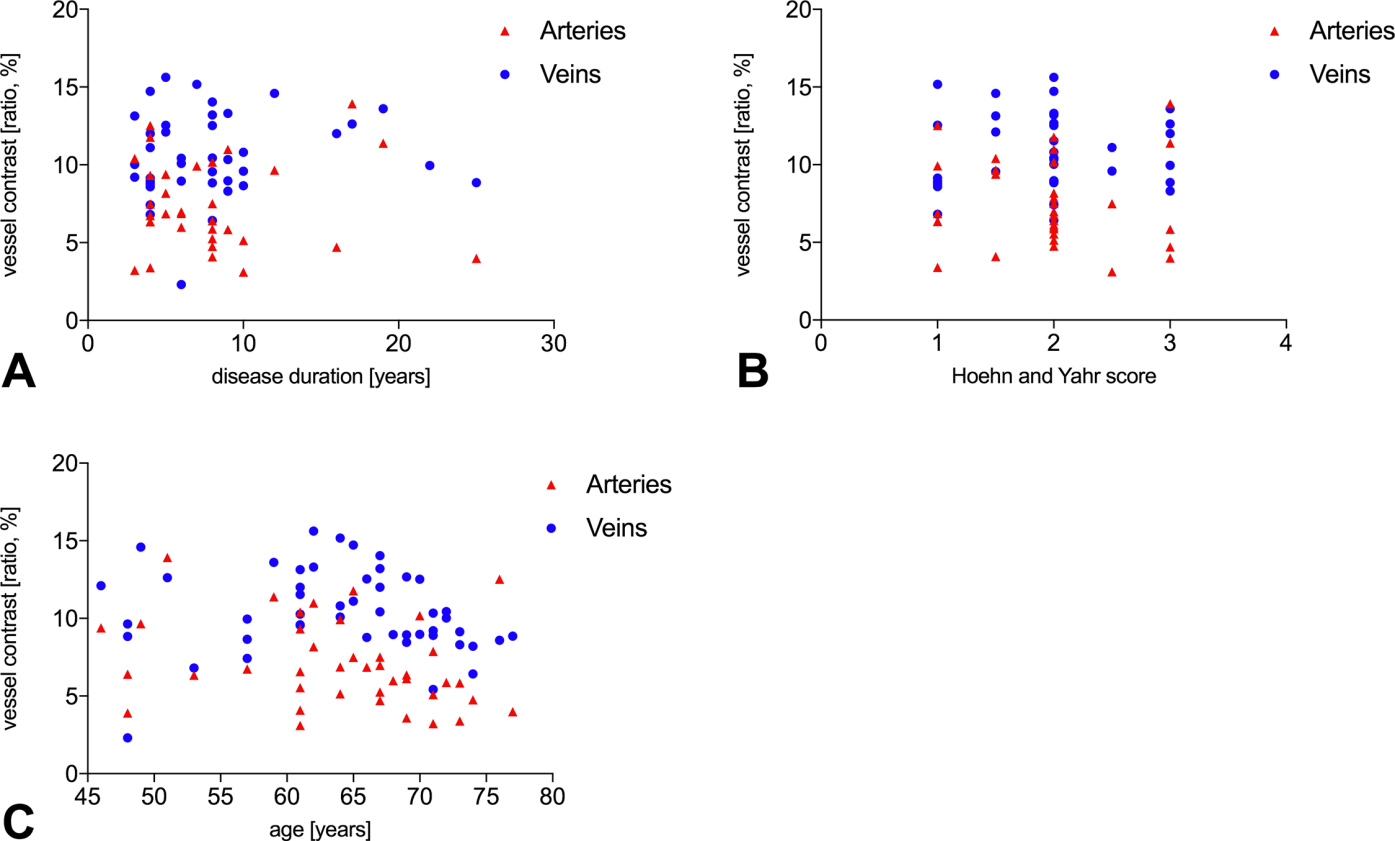
Correlation of the vessel contrast change in patients with PD with parameters of disease progression and age. (A) Disease duration did not correlate with the vessel contrast (*p*-value>0.05). (B) The clinical progression chart Hoehn and Yahr did not correlate with the vessel contrast (*p*-value>0.05). (C) The age of the patients did not correlate with the vessel contrast (*p*-value>0.05).

Disease duration, disease severity according to Hoehn and Yahr or age did not correlate with the grey contrast between vessels and the surrounding tissue (*p*-value>0.05, see Fig 4).

**Fig 5:**
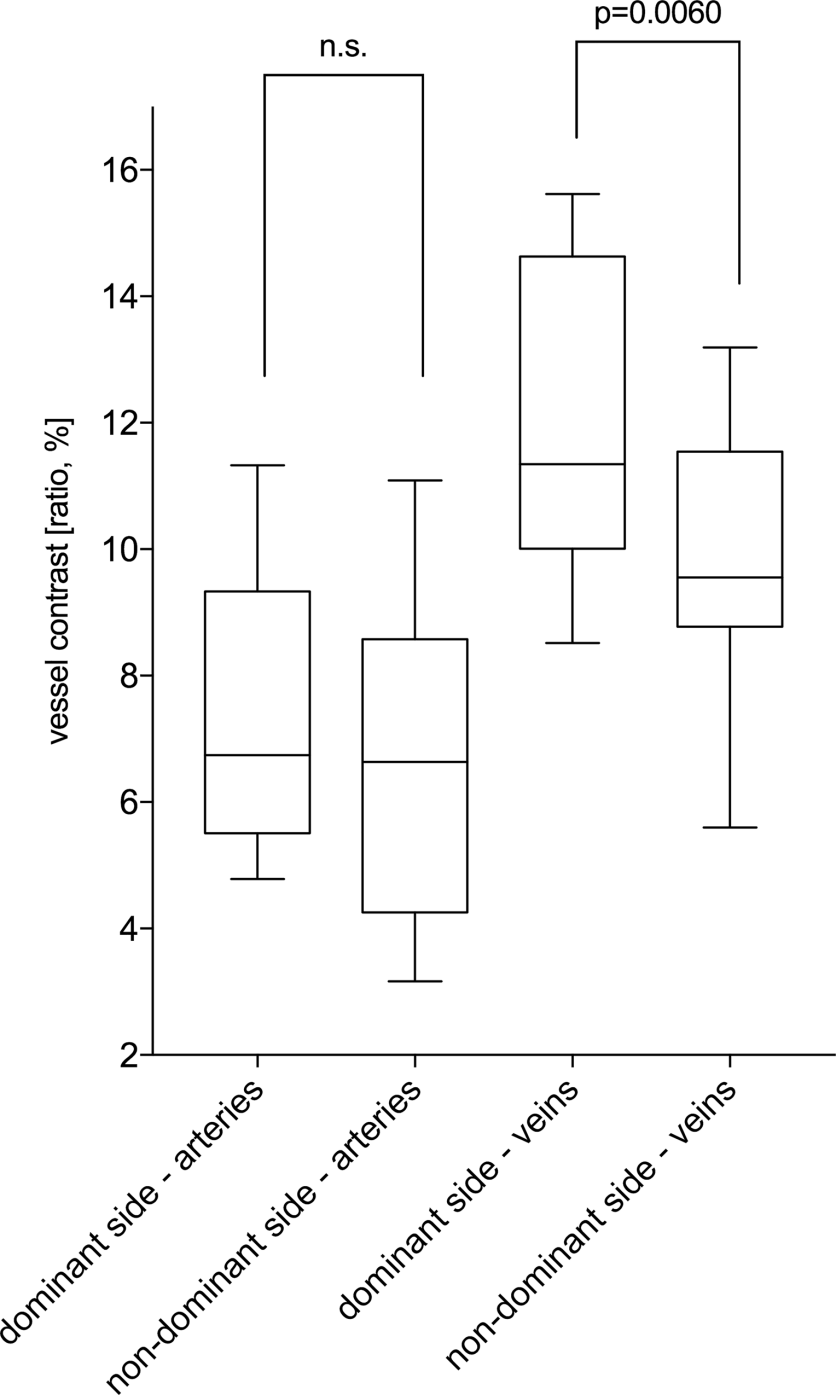
Comparison of the vessel contrast change between the dominant and non-dominant side of PD patients. The grey value vessel ratio for veins was significantly different (*p*-value = 0.0060), while arteries showed no significant difference (*p*-value>0.05).

The inter-individual comparison in PD patients between the clinical dominant and non-dominant side showed a highly significant difference of the grey value vessel ratio for veins (*p*-value = 0.0060): The non-dominant side showed a lower contrast in veins compared to the dominant side. The arteries showed no significant difference for this comparison (*p*-value>0.05) (Fig 5).

## Discussion

In this study, we assessed retinal vessel morphology in PD patients using a novel technique of spectral domain—optical coherence tomography (SD-OCT) application which allows investigating the signal strength of grey values and vessel diameter as parameters for changes in vessel morphology. Our data indicates altered retinal vasculature in PD patients compared to controls mainly due to changes of retinal veins. In PD patients the similarity between the grey value within the venous lumen and its surrounding tissue (smaller grey vessel contrast) could be the result of reduced perfusion, but changes in the vessel walls or blood velocity are also possible causes.

There is increasing interest in vessel analysis in neurological disorders. Retinal imaging modalities other than OCT have been used to study especially small vessel disease (SVD) due to both cerebrovascular diseases and systemic disorders such as hypertension, cerebral hypoperfusion, inflammation, and vascular dementia [34]. Small vessel pathology also seems to play a role in primary neurodegenerative disorders and co-occurrence of central neurodegenerative and vascular pathologies with SVD have been found on a regular basis histopathologically in late-onset Alzheimer’s Disease (AD) [35].

In recent years OCT has been increasingly applied to assess structural retinal changes as a result of tissue damage, retinal dysfunction or degenerative changes in neurological diseases. OCT allows in vivo imaging of tissue morphology with much higher resolution than other imaging techniques such as MRI or ultrasound [14]. It has been used in multiple sclerosis, neuro-ophthalmic diseases, and latterly in central neurodegenerative diseases such as PD, amyotrophic lateral sclerosis, and AD [36].

SD-OCT as further development of OCT has been used recently to detect SVD in cerebral autosomal dominant arteriopathy with subcortical infarcts and leukoencephalopathy (CADASIL) [37] and might become a diagnostic and prognostic complementary tool in the future to assess retinal vessel changes in patients suffering from cerebral small-vessel diseases (SVD) such as vascular cognitive impairment.

In this study, we introduce a novel technical application using SD-OCT to analyse retinal vessel morphology in PD patients. Parkinson´s disease is supposed to be primarily a neurodegenerative disease. However, despite inconclusive data results of the majority of neuropathological studies suggest that PD patients possibly have an increased risk of developing comorbid cerebrovascular disease compared with the general population [15]. Striatal lacunar infarction was found to be 3 times more common than cortical infarction in pathologically confirmed PD [38] suggesting small vessel disease (SVD) as predominant stroke subtype in PD [16]. A recent prospective study using different magnetic resonance imaging (MRI) and diffusion tensor imaging (DTI) measurements showed cerebral SVD to be associated with incident parkinsonism, suggesting a role of SVD in the aetiology of parkinsonism [17].

Furthermore, there is some argument that SVD in PD patients potentially is related to chronic levodopa therapy. It is known that after a disease duration of 4 to 5 years, 66 to 72% of PD patients who initially start therapy without levodopa, add or change to levodopa treatment [39,40]. Levodopa therapy is associated with increased plasma levels of homocysteine [41], which is known to correlate with generalised SVD [42] including cerebral SVD [42,43]. Therefore, SVD might be the underlying neurotoxic pathology for polyneuropathy in PD patients with chronic levodopa therapy [18–22]. However, hyperhomocysteinemia is assumed to be a risk factor not only for vascular and neurodegenerative diseases but also ocular diseases such as retinopathy, pseudoexfoliative glaucoma, maculopathy, cataract, optic atrophy and retinal vessel atherosclerosis due to impaired vascular endothelial function [44]. Patients in our study had a relatively long disease duration [median 8 years; range 2–24 years] and most patients were treated with levodopa. Therefore, SVD might be causal for our findings of a reduced grey value vessel ratio in PD.

However, we found retinal veins but not arteries being responsible for the lower grey vessel value contrast in PD patients compared to controls. This was unexpected and seems to be somewhat surprisingly at first sight because SVD in PD likely is related to arteries more than to veins. On the other hand, it is well known that venous retinal pathology results from arteriosclerotic diseases such as arterial hypertension, diabetes mellitus or hyperlipidemia in patients with retinal vein occlusions (branch and central retinal vein occlusions) [45]. It is widely accepted that arterial rigidity and turbulent flows affect the retinal vein lying next to the artery and the capillary network. Vein and artery often share the adventitial sheath and the thicker and stiffer arterial wall can compromise the venous lumen und blood flow [46]. So in retinal vein occlusion, retinal venous changes are a result of preceding changes in the arterial branch of circulation. We suspect this to be the case in our PD patients, too. It is possible that veins are an even more sensitive parameter for SVD in PD than arterial retinal pathology. It is also conceivable that our method yields better and more precise results in the venous branch of circulation because first, we were able to detect more veins per PD patient or control in the OCT scans and second veins could be measured more easily because of their relatively three times bigger lumen compared to arteries.

Further studies should investigate the relevance of arterial and venous retinal changes in PD and also assess a potential relationship between cumulative levodopa doses, homocysteine plasma levels and grey value arterial and venous vessel ratio in PD patients.

We found retinal vessel changes to be more prominent contralateral to the clinically predominantly affected body side in the PD patients. This is in line with the characteristic asymmetric neurodegenerative process in PD patients with higher nigrostriatal dopaminergic deficit contralateral to the predominantly motor-impaired body side [47,48] and also with the recent finding of olfactory asymmetric dysfunction in early PD patients with the unilateral disorder [49].

We did not find the dependence of disease duration or disease severity with retinal vessel changes. This is in line with results of some previous OCT measures, showing no association of RNFL, total macular volume, or intra-retinal layer segmentation with disease severity or duration [50] but not with other RFNL studies in PD [51]. Large studies with a greater sample size are needed to obtain statistically reliable associations regarding changes of retinal vessel architecture and clinical features of PD patients.

Noteworthy, vascular pathology as underlying contributive etiopathogenetic process has been well accepted for normal tension glaucoma (NTG) as another primarily neurodegenerative ocular disease. There are several similarities of changes in patients with normal tension glaucoma (NTG) and PD. First, as known for NTG [52], progressive neurodegeneration of the RNFL has also been described in PD [12]. Second, optic neuropathy is the disease-defining factor in NTG and is suspected to play a role in PD [53,54]. Third, perfusion of the optic disc is grossly reduced in NTG [55] and likely also in PD patients, because the optic nerve in PD patients has been found to show increased paleness as a result of decreased haemoglobin levels [56]. Finally, variations in circadian blood pressure with either a missing (“non-dipper”) or an enhanced (“over-dipper”) physiologic dip during the night are common in NTG [57] and PD patients [58]. Therefore, it is possible that vascular changes play a role in the degenerative processes within the retina and clinical visual symptoms in both NTG and PD.

Our method of high-resolution SD-OCT-based vessel analysis works semi-automatically, enables results that are mainly independent of the observer and yields the possibility of near histological representation of tissue. The specific grey value within the vessel lumen of each patient is likely influenced by the flow speed of the blood, the viscosity of the blood and by changes in the vessel walls. The grey value can be measured precisely for each vessel and averaged for the patient. Influences of optic media opacities or image brightness are not relevant because the grey value is compared to the directly surrounding tissue and not to an absolute value. However, due to different scattering properties of the tissue, it might appear different than under the microscope. In contrast, other current methods for evaluating retinal vasculature, such as manual or automatic analysis of visible vessels in fundus photography [59,60] and confocal scanning laser ophthalmoscopy (cSLO) [30] often do not allow evaluation of retinal vessel morphology in great detail. They are also more observer dependent and therefore show a large inter-observer variability in assessing the retinal vasculature. For these reasons, our method might better fulfil the demand for an objective measurement of retinal vessel morphology [61].

Our study has some limitations: First, we were not able to differentiate between arteries and veins completely automatically. Automatic labelling vessels in SD-OCT seems to be more complicated compared to conventional fundus photography and is still under discussion [30]. Second, we did not record the cumulative levodopa dose and the homocysteine plasma levels and therefore could not correlate findings of retinal vessel morphology with these parameters, potentially influencing vascular retinal morphology.

The strengths of our study are (i) a comparably large sample size, (ii) a standardised manufacturer-proposed acquisition technique, and (iii) the use of a semi-automated approach with quality control. As far as we know, a comparison of PD patients to healthy controls concerning retinal perfusion has not been previously published.

In summary, we have demonstrated that it is possible to assess retinal vasculature using standardised and non-invasive SD-OCT technology. Our findings demonstrate that there might be changes in retinal venous vessel morphology and perfusion in PD patients compared with age- and sex-matched healthy controls, potentially as a result of small vessel disease in PD. Altered retinal vasculature in PD might contribute to a degenerative process within the retina and to clinical symptoms, such as reading difficulties, diminished colour or contrast vision or diplopia. Longitudinal studies with larger sample sizes are needed to assess the ability of our approach to providing a new biomarker of neurodegeneration and vasculopathy in PD and other neurodegenerative diseases.

## Supporting Information

S1 Table Complete raw data. (XLSX)

## Abbreviations

PD: Parkinson’s disease
RNFL: retinal nerve fibre layer
cSLO: confocal scanning laser ophthalmoscopy
SD-OCT: spectral domain optical coherence tomography
GCL: ganglion cell layer
ILM: internal limiting membrane
NTG: normal-tension glaucoma

## References

[1] Braak H, Del Tredici K, Bratzke H, Hamm-Clement J, Sandmann-Keil D, Rüb U. Staging of the intracerebral inclusion body pathology associated with idiopathic Parkinson's disease (preclinical and clinical stages). J Neurol. 2002;249 Suppl 3: III–1–5. 10.1007/s00415-002-1301-4 12528692

[2] Hawkes CH, Del Tredici K, Braak H. A timeline for Parkinson's disease. Parkinsonism Relat Disord. 2010;16: 79–84. 10.1016/j.parkreldis.2009.08.007 19846332

[3] Archibald NK, Clarke MP, Mosimann UP, Burn DJ. The retina in Parkinson's disease. Brain. Oxford University Press; 2009;132: 1128–1145. 10.1093/brain/awp068 19336464

[4] Chaudhuri KR, Healy DG, Schapira AHV, National Institute for Clinical Excellence. Non-motor symptoms of Parkinson's disease: diagnosis and management. Lancet Neurol. 2006;5: 235–245. 10.1016/S1474-4422(06)70373-8 16488379

[5] Liew G, Wang JJ, Mitchell P, Wong TY. Retinal vascular imaging: a new tool in microvascular disease research. Circ Cardiovasc Imaging. Lippincott Williams & Wilkins; 2008;1: 156–161. 10.1161/CIRCIMAGING.108.784876 19808533

[6] Aaker GD, Myung JS, Ehrlich JR, Mohammed M, Henchcliffe C, Kiss S. Detection of retinal changes in Parkinson's disease with spectral-domain optical coherence tomography. Clin Ophthalmol. Dove Press; 2010;4: 1427–1432. 10.2147/OPTH.S15136 21188154 PMC3000768

[7] Albrecht P, Müller A-K, Südmeyer M, Ferrea S, Ringelstein M, Cohn E, et al Optical coherence tomography in parkinsonian syndromes. Paul F, editor. PLoS ONE. Public Library of Science; 2012;7: e34891 10.1371/journal.pone.0034891 22514688 PMC3325949

[8] Ding Y, Spund B, Glazman S, Shrier EM, Miri S, Selesnick I, et al Application of an OCT data-based mathematical model of the foveal pit in Parkinson disease. J Neural Transm (Vienna). Springer Vienna; 2014;121: 1367–1376. 10.1007/s00702-014-1214-2 24748549

[9] Garcia-Martin E, Satue M, Fuertes I, Otin S, Alarcia R, Herrero R, et al Ability and reproducibility of Fourier-domain optical coherence tomography to detect retinal nerve fiber layer atrophy in Parkinson's disease. Ophthalmology. 2012;119: 2161–2167. 10.1016/j.ophtha.2012.05.003 22749083

[10] Hajee ME, March WF, Lazzaro DR, Wolintz AH, Shrier EM, Glazman S, et al Inner retinal layer thinning in Parkinson disease. Arch Ophthalmol. American Medical Association; 2009;127: 737–741. 10.1001/archophthalmol.2009.106 19506190

[11] Bittersohl D, Stemplewitz B, Keserü M, Buhmann C, Richard G, Hassenstein A. Detection of retinal changes in idiopathic Parkinson's disease using high-resolution optical coherence tomography and heidelberg retina tomography. Acta Ophthalmologica. 2015;93: e578–84. 10.1111/aos.12757 26267660

[12] Stemplewitz B, Keserü M, Bittersohl D, Buhmann C, Skevas C, Richard G, et al Scanning laser polarimetry and spectral domain optical coherence tomography for the detection of retinal changes in Parkinson's disease. Acta Ophthalmologica. 2015;93: n/a–n/a. 10.1111/aos.12764 26066643

[13] Schneider M, Müller H-P, Lauda F, Tumani H, Ludolph AC, Kassubek J, et al Retinal single-layer analysis in Parkinsonian syndromes: an optical coherence tomography study. J Neural Transm (Vienna). Springer Vienna; 2014;121: 41–47. 10.1007/s00702-013-1072-3 23907408

[14] Lee J-Y, Ahn J, Kim TW, Jeon BS. Optical coherence tomography in Parkinson's disease: is the retina a biomarker? J Parkinsons Dis. IOS Press; 2014;4: 197–204. 10.3233/JPD-130306 24518436

[15] Nanhoe-Mahabier W, de Laat KF, Visser JE, Zijlmans J, de Leeuw F-E, Bloem BR. Parkinson disease and comorbid cerebrovascular disease. Nat Rev Neurol. Nature Publishing Group; 2009;5: 533–541. 10.1038/nrneurol.2009.136 19724249

[16] Schwartz RS, Halliday GM, Cordato DJ, Kril JJ. Small-vessel disease in patients with Parkinson's disease: a clinicopathological study. Mov Disord. Wiley Subscription Services, Inc., A Wiley Company; 2012;27: 1506–1512. 10.1002/mds.25112 23015464

[17] van der Holst HM, van Uden IWM, Tuladhar AM, de Laat KF, van Norden AGW, Norris DG, et al Cerebral small vessel disease and incident parkinsonism: The RUN DMC study. Neurology. Lippincott Williams & Wilkins; 2015;85: 1569–1577. 10.1212/WNL.0000000000002082 26446068 PMC4642143

[18] Kimber T, Blumbergs P, Thompson P. Severe ataxic polyneuropathy associated with chronic levodopa use in Parkinson's disease. Parkinsonism Relat Disord. 2013;19: 847–849. 10.1016/j.parkreldis.2013.05.002 23746939

[19] Shahrizaila N, Mahamad UA, Yap A-C, Choo Y-M, Marras C, Lim S-Y. Is chronic levodopa therapy associated with distal symmetric polyneuropathy in Parkinson's disease? Parkinsonism Relat Disord. 2013;19: 391–393. 10.1016/j.parkreldis.2012.08.002 23146348

[20] Nolano M, Provitera V, Lanzillo B, Santoro L. Neuropathy in idiopathic Parkinson disease: an iatrogenic problem? Ann Neurol. 2011;69: 427–8– author reply 428–9. 10.1002/ana.22330 21387392

[21] Montastruc J-L, Danton AC, Durrieu G, Lacroix I, Olivier P, Sommet A, et al Neuropathy as a potential complication of levodopa use in Parkinson's disease: a pharmacological and pharmacovigilance point of view. Mov Disord. 2010;25: 660–661. 10.1002/mds.22878 20213824

[22] Merola A, Romagnolo A, Zibetti M, Bernardini A, Cocito D, Lopiano L. Peripheral neuropathy associated with levodopa-carbidopa intestinal infusion: a long-term prospective assessment. Eur J Neurol. 2016;23: 501–509. 10.1111/ene.12846 26498913

[23] Wojtkowski M, Bajraszewski T, Targowski P, Kowalczyk A. Real-time in vivo imaging by high-speed spectral optical coherence tomography. Opt Lett. 2003;28: 1745–1747. 14514087 10.1364/ol.28.001745

[24] Cense B, Nassif N, Chen T, Pierce M, Yun S-H, Park B, et al Ultrahigh-resolution high-speed retinal imaging using spectral-domain optical coherence tomography. Opt Express. 2004;12: 2435–2447. 10.1364/OPEX.12.002435 19475080

[25] Lee P, Gao W, Zhang X. Performance of single-scattering model versus multiple-scattering model in the determination of optical properties of biological tissue with optical coherence tomography. Appl Opt. 2010;49: 3538–3544. 10.1364/AO.49.003538 20563206

[26] Hughes AJ, Daniel SE, Kilford L, Lees AJ. Accuracy of clinical diagnosis of idiopathic Parkinson's disease: a clinico-pathological study of 100 cases. J Neurol Neurosurg Psychiatr. 1992;55: 181–184. 10.1136/jnnp.55.3.181 1564476 PMC1014720

[27] Hoehn MM, Yahr MD. Parkinsonism: onset, progression and mortality. Neurology. 1967;17: 427–442. 6067254 10.1212/wnl.17.5.427

[28] Tewarie P, Balk L, Costello F, Green A, Martin R, Schippling S, et al The OSCAR-IB consensus criteria for retinal OCT quality assessment. Villoslada P, editor. PLoS ONE. Public Library of Science; 2012;7: e34823 10.1371/journal.pone.0034823 22536333 PMC3334941

[29] Lu S, Cheung CYL, Liu J, Lim JH, Leung CKS, Wong TY. Automated layer segmentation of optical coherence tomography images. IEEE Trans Biomed Eng. 2010;57: 2605–2608. 10.1109/TBME.2010.2055057 20595078

[30] Motte J, Alten F, Ewering C, Osada N, Kadas EM, Brandt AU, et al Vessel labeling in combined confocal scanning laser ophthalmoscopy and optical coherence tomography images: criteria for blood vessel discrimination. PLoS ONE. 2014;9: e102034 10.1371/journal.pone.0102034 25203135 PMC4159183

[31] Staurenghi G, Sadda S, Chakravarthy U, Spaide RF, International Nomenclature for Optical Coherence Tomography (IN•OCT) Panel. Proposed lexicon for anatomic landmarks in normal posterior segment spectral-domain optical coherence tomography: the IN•OCT consensus. 2014. pp. 1572–1578. doi: 10.1016/j.ophtha.2014.02.023 24755005

[32] Herzog A, Boyer KL, Roberts C. Robust Extraction of the Optic Nerve Head in Optical Coherence Tomography Computer Vision and Mathematical Methods in Medical and Biomedical Image Analysis. Berlin, Heidelberg: Springer Berlin Heidelberg; 2004 pp. 395–407. 10.1007/978-3-540-27816-0_34

[33] Yang Q, Reisman CA, Wang Z, Fukuma Y, Hangai M, Yoshimura N, et al Automated layer segmentation of macular OCT images using dual-scale gradient information. Opt Express. 2010;18: 21293–21307. 10.1364/OE.18.021293 20941025 PMC3101081

[34] de Jong FJ, Schrijvers EMC, Ikram MK, Koudstaal PJ, de Jong PTVM, Hofman A, et al Retinal vascular caliber and risk of dementia: the Rotterdam study. Neurology. Lippincott Williams & Wilkins; 2011;76: 816–821. 10.1212/WNL.0b013e31820e7baa 21288987 PMC3053331

[35] Marnane M, Hsiung G-YR. Could Better Phenotyping Small Vessel Disease Provide New Insights into Alzheimer Disease and Improve Clinical Trial Outcomes? Curr Alzheimer Res. 2016;13: 750–763. 26899579 10.2174/1567205013666160222112634

[36] Gupta S, Zivadinov R, Ramanathan M, Weinstock-Guttman B. Optical coherence tomography and neurodegeneration: are eyes the windows to the brain? Expert Rev Neurother. 2016;16: 765–775. 10.1080/14737175.2016.1180978 27138997

[37] Alten F, Motte J, Ewering C, Osada N, Clemens CR, Kadas EM, et al Multimodal retinal vessel analysis in CADASIL patients. Stieger K, editor. PLoS ONE. 2014;9: e112311 10.1371/journal.pone.0112311 25372785 PMC4221286

[38] Hughes AJ, Daniel SE, Blankson S, Lees AJ. A clinicopathologic study of 100 cases of Parkinson's disease. Arch Neurol. 1993;50: 140–148. 8431132 10.1001/archneur.1993.00540020018011

[39] Parkinson Study Group. Pramipexole vs levodopa as initial treatment for Parkinson disease: A randomized controlled trial. Parkinson Study Group. JAMA. 2000;284: 1931–1938. 11035889 10.1001/jama.284.15.1931

[40] Rascol O, Brooks DJ, Korczyn AD, De Deyn PP, Clarke CE, Lang AE. A five-year study of the incidence of dyskinesia in patients with early Parkinson's disease who were treated with ropinirole or levodopa. N Engl J Med. 2000;342: 1484–1491. 10.1056/NEJM200005183422004 10816186

[41] Müller T, Woitalla D, Fowler B, Kuhn W. 3-OMD and homocysteine plasma levels in parkinsonian patients. J Neural Transm (Vienna). 2002;109: 175–179. 10.1007/s007020200013 12075857

[42] Kloppenborg RP, Geerlings MI, Visseren FL, Mali WPTM, Vermeulen M, van der Graaf Y, et al Homocysteine and progression of generalized small-vessel disease: the SMART-MR Study. Neurology. 2014;82: 777–783. 10.1212/WNL.0000000000000168 24477110

[43] Hassan A, Hunt BJ, O’Sullivan M, Bell R, D’Souza R, Jeffery S, et al Homocysteine is a risk factor for cerebral small vessel disease, acting via endothelial dysfunction. Brain. 2004;127: 212–219. 10.1093/brain/awh023 14607791

[44] Ajith TA, Ranimenon. Homocysteine in ocular diseases. Clin Chim Acta. 2015;450: 316–321. 10.1016/j.cca.2015.09.007 26343924

[45] Kolar P. Risk factors for central and branch retinal vein occlusion: a meta-analysis of published clinical data. J Ophthalmol. Hindawi Publishing Corporation; 2014;2014: 724780–5. 10.1155/2014/724780 25009743 PMC4070325

[46] Rehak J, Rehak M. Branch retinal vein occlusion: pathogenesis, visual prognosis, and treatment modalities. Curr Eye Res. Taylor & Francis; 2008;33: 111–131. 10.1080/02713680701851902 18293182 PMC2430176

[47] Tatsch K, Schwarz J, Mozley PD, Linke R, Pogarell O, Oertel WH, et al Relationship between clinical features of Parkinson's disease and presynaptic dopamine transporter binding assessed with [123I]IPT and single-photon emission tomography. Eur J Nucl Med. 1997;24: 415–421. 9096093 10.1007/BF00881814

[48] Pirker W. Correlation of dopamine transporter imaging with parkinsonian motor handicap: how close is it? Mov Disord. Wiley Subscription Services, Inc., A Wiley Company; 2003;18 Suppl 7: S43–51. 10.1002/mds.10579 14531046

[49] Zucco GM, Rovatti F, Stevenson RJ. Olfactory asymmetric dysfunction in early Parkinson patients affected by unilateral disorder. Front Psychol. Frontiers; 2015;6: 1020 10.3389/fpsyg.2015.01020 26236275 PMC4503885

[50] Roth NM, Saidha S, Zimmermann H, Brandt AU, Isensee J, Benkhellouf-Rutkowska A, et al Photoreceptor layer thinning in idiopathic Parkinson's disease. Mov Disord. 2014;29: 1163–1170. 10.1002/mds.25896 24789530

[51] Jiménez B, Ascaso FJ, Cristóbal JA, López del Val J. Development of a prediction formula of Parkinson disease severity by optical coherence tomography. Mov Disord. 2014;29: 68–74. 10.1002/mds.25747 24458320

[52] Jaffe GJ, Caprioli J. Optical coherence tomography to detect and manage retinal disease and glaucoma. Am J Ophthalmol. 2004;137: 156–169. 10.1016/S0002-9394(03)00792-X 14700659

[53] La Morgia C, Barboni P, Rizzo G, Carbonelli M, Savini G, Scaglione C, et al Loss of temporal retinal nerve fibers in Parkinson disease: a mitochondrial pattern? Eur J Neurol. Blackwell Publishing Ltd; 2013;20: 198–201. 10.1111/j.1468-1331.2012.03701.x 22436028

[54] Maresca A, la Morgia C, Caporali L, Valentino ML, Carelli V. The optic nerve: a “mito-window” on mitochondrial neurodegeneration. Mol Cell Neurosci. 2013;55: 62–76. 10.1016/j.mcn.2012.08.004 22960139 PMC3629569

[55] Chung HS, Harris A, Kagemann L, Martin B. Peripapillary retinal blood flow in normal tension glaucoma. British Journal of Ophthalmology. BMJ Group; 1999;83: 466–469. 10434872 10.1136/bjo.83.4.466 PMC1722989

[56] Bambo MP, Garcia-Martin E, Satue M, Perez-Olivan S, Alayon S, Gonzalez-Hernandez M, et al Measuring hemoglobin levels in the optic disc of Parkinson's disease patients using new colorimetric analysis software. Parkinsons Dis. 2014;2014: 946540–946548. 10.1155/2014/946540 25587487 PMC4284935

[57] Choi J, Jeong J, Cho H-S, Kook MS. Effect of nocturnal blood pressure reduction on circadian fluctuation of mean ocular perfusion pressure: a risk factor for normal tension glaucoma. Invest Ophthalmol Vis Sci. The Association for Research in Vision and Ophthalmology; 2006;47: 831–836. 10.1167/iovs.05-1053 16505014

[58] Berganzo K, Díez-Arrola B, Tijero B, Somme J, Lezcano E, Llorens V, et al Nocturnal hypertension and dysautonomia in patients with Parkinson's disease: are they related? J Neurol. Springer Berlin Heidelberg; 2013;260: 1752–1756. 10.1007/s00415-013-6859-5 23412356

[59] DeBuc DC. A Review of Algorithms for Segmentation of Retinal Image Data Using Optical Coherence Tomography In: Ho P-GP, editor. Image Segmentation. InTech; 2011.

[60] Fraz MM, Remagnino P, Hoppe A, Uyyanonvara B, Rudnicka AR, Owen CG, et al Blood vessel segmentation methodologies in retinal images—a survey. Computer Methods and Programs in Biomedicine. 2012;108: 407–433. 10.1016/j.cmpb.2012.03.009 22525589

[61] Kwa VIH, van der Sande JJ, Stam J, Tijmes N, Vrooland JL, Amsterdam Vascular Medicine Group. Retinal arterial changes correlate with cerebral small-vessel disease. Neurology. 2002;59: 1536–1540. 12451193 10.1212/01.wnl.0000033093.16450.5c

